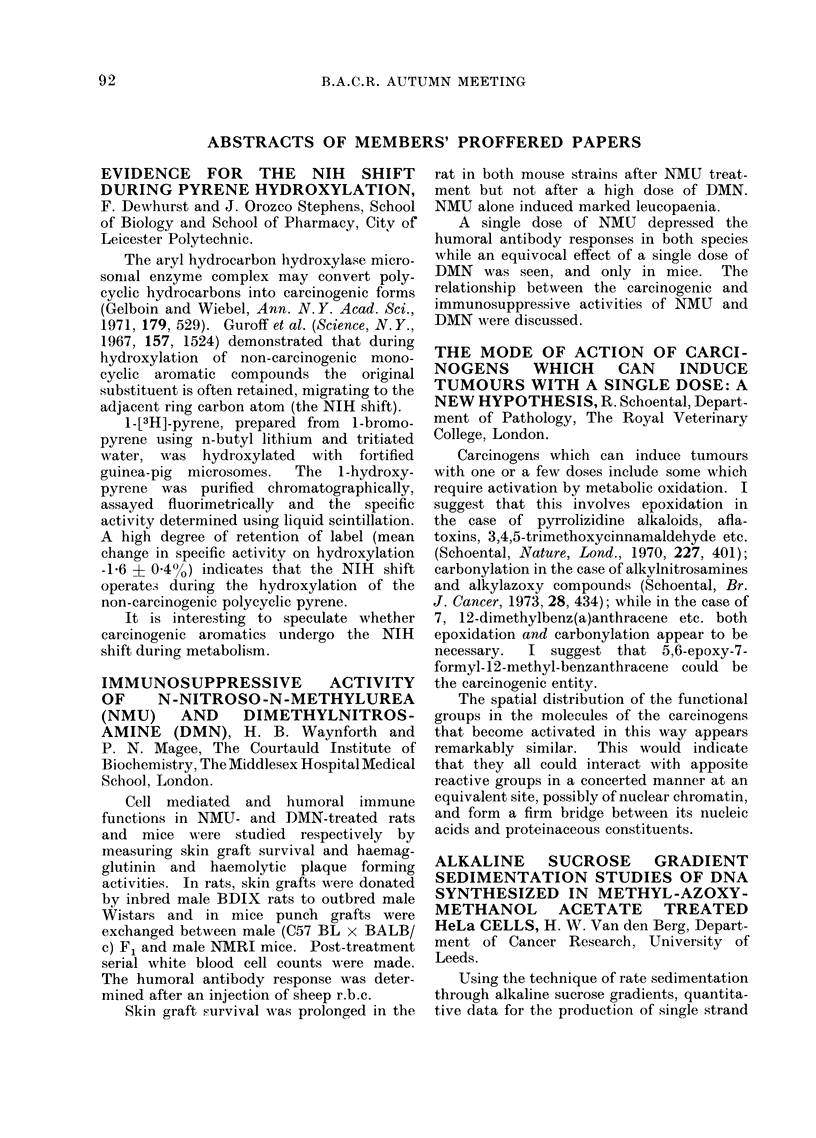# Proceedings: Evidence for the NIH shift during pyrene hydroxylation.

**DOI:** 10.1038/bjc.1974.13

**Published:** 1974-01

**Authors:** F. Dewhurst, J. O. Stephens


### ABSTRACTS OF MEMBERS' PROFFERED PAPERS

**EVIDENCE FOR THE NIH SHIFT DURING PYRENE HYDROXYLATION,** F. Dewhurst and J. Orozco Stephens, School of Biology and School of Pharmacy, City of Leicester Polytechnic.

The aryl hydrocarbon hydroxylase microsomal enzyme complex may convert polycyclic hydrocarbons into carcinogenic forms (Gelboin and Wiebel, *Ann. N.Y. Acad. Sci.*, 1971, **179**, 529). Guroff *et al.* (*Science, N.Y.*, 1967, **157**, 1524) demonstrated that during hydroxylation of non-carcinogenic monocyclic aromatic compounds the original substituent is often retained, migrating to the adjacent ring carbon atom (the NIH shift).

1-[$^3$H]-pyrene, prepared from 1-bromopyrene using n-butyl lithium and tritiated water, was hydroxylated with fortified guinea-pig microsomes. The 1-hydroxypyrene was purified chromatographically, assayed fluorimetrically and the specific activity determined using liquid scintillation. A high degree of retention of label (mean change in specific activity on hydroxylation $-1{\cdot}6 \pm 0{\cdot}4\%$) indicates that the NIH shift operates during the hydroxylation of the non-carcinogenic polycyclic pyrene.

It is interesting to speculate whether carcinogenic aromatics undergo the NIH shift during metabolism.